# Human Hand Motion Analysis during Different Eating Activities

**DOI:** 10.1155/2018/8567648

**Published:** 2018-02-04

**Authors:** Zakia Hussain, Norsinnira Zainul Azlan, Arif Zuhairi bin Yusof

**Affiliations:** Department of Mechatronics Engineering, International Islamic University Malaysia, Gombak, 53100 Kuala Lumpur, Malaysia

## Abstract

The focus of this research is to analyse both human hand motion and force, during eating, with respect to differing food characteristics and cutlery (including a fork and a spoon). A glove consisting of bend and force sensors has been used to capture the motion and contact force exerted by fingers during different eating activities. The Pearson correlation coefficient has been used to show that a significant linear relationship exists between the bending motion of the fingers and the forces exerted during eating. Analysis of variance (ANOVA) and independent samples *t*-tests are performed to establish whether the motion and force exerted by the fingers while eating is influenced by the different food characteristics and cutlery. The middle finger motion showed the least positive correlation with index fingertip and thumb-tip force, irrespective of the food characteristics and cutlery used. The ANOVA and *t*-test results revealed that bending motion of the index finger and thumb varies with respect to differing food characteristics and the type of cutlery used (fork/spoon), whereas the bending motion of the middle finger remains unaffected. Additionally, the contact forces exerted by the thumb tip and index fingertip remain unaffected with respect to differing food types and cutlery used.

## 1. Introduction

Upper limb disability is one of the major adversities faced by poststroke patients. The resulting loss of mobility in these patients reduces their ability to perform normal activities of daily living (ADL), preventing them from leading a normal life and hence reducing their quality of life. These patients are highly dependent on their caregivers (usually a spouse or friend) who perform most of their basic ADL, such as eating, bathing, and grooming, which gradually has a negative impact on the mental and physical state of the caregiver [1–6].

Eating is one of the fundamental activities of survival for all living beings. Dysphagia and other eating difficulties are also common among poststroke patients which can lead to complications, such as malnutrition, dehydration, suffocation, and eventually death [7–10]. Over the past decade, numerous robotic rehabilitation systems have been developed to assist impaired patients regain their hand functions. Such robotic systems must have the capability to replicate human hand function during any ADL. To develop a rehabilitation system meant specifically to regain the hand function during eating, an in-depth knowledge of hand motion during eating is vital.

Hand motion during eating is highly dexterous and is subject to the type of food ingested and the type of cutlery used. Analysing hand motion can be complicated due to its highly articulate nature. A human hand consists of 27 bones and 35 muscles, of which 17 are intrinsic muscles (located in the palm) and 18 are extrinsic muscles (located in the forearm). With roughly 30 degrees of freedom (DOFs), this complex structure can perform intricate tasks, which require dexterity. During the past few years, hand motion analysis has gained the attention of the researchers working in the field of rehabilitation, human-computer interaction (HCI), and robotics.

Hand motion analysis enables researchers to gather data such as the force applied by the fingers, different joint angles of the hand, and velocity, while performing different activities. Analysing the motion and force of the hand during various eating activities can help in formulating a model, which in turn can be useful in developing a rehabilitation robot for assisted eating. Several studies have been conducted to analyse the motion of the hand and upper limb while performing different daily activities of living. Ju and Liu [11], Gopura et al. [12], and Tang et al. [13] have successfully analysed and classified different human hand motions while performing basic daily activities, such as hair combing and recognizing multiple hand gestures, using electromyography (EMG). In EMG analysis, tiny electrodes, when placed on human skin, detect and record the electrical signals transmitted by the motor neurons responsible for activating muscle contraction. Ju and Liu [11] used a framework of multiple sensor integration of CyberGlove, Finger TPS pressure sensors, and Trigno wireless EMG sensors to capture hand gestures, contact forces, and muscle contraction signals from various hand motions, while performing 10 basic grasping activities, such as holding and lifting a dumbbell and opening and closing a pen box, using five fingers.

Cabibihan et al. [14] explored the human patting gesture for analysing the amount of force applied to regions of the hand and the angular motion of finger joints so as to incorporate them into a humanoid robot, in order to imitate this gesture. Similarly, the kinematics and dynamics of the human arm, during 24 daily activities (such as eating using a spoon and a fork, drinking with a cup, and washing the face) were studied by Rosen et al. [15] to develop a 7-DOF powered exoskeleton for the upper limb. Ah et al. [16] performed human hand motion analysis while turning a door knob.

Aprile et al. [17] dedicated an entire study to analyse the upper limb motion in stroke patients while performing a drinking task, which included reaching for the glass, bringing it to the mouth, and putting it back on the table. Adnan et al. [18] developed a low-cost DataGlove using a flexible bend sensor to recognize various human finger activities. In addition, the analytical mathematical model and analysis of variance (ANOVA) was established to predict the force induced at the flexible force sensor by the human finger using the low-cost DataGlove [19].

Some previous work on hand analysis is summarized in Table 1. Despite many studies on hand motion (Table 1), to the best of our knowledge, there has not been a study dedicated to the analysis of hand motion while eating different types of food and using different cutlery. It is important to consider the food characteristics and the amount of force exerted by the hand during eating to enhance the development of robotic rehabilitation systems for this activity.

Therefore, this paper presents human hand motion analysis, focusing on the thumb, index finger, and middle finger during eating. The motion of these three fingers and the force they exert during eating is studied with respect to the type of food (liquid, solid) and the cutlery used. An experiment has been conducted involving five different food types and using two different types of cutlery (fork and spoon) to study their effect on hand motion. ANOVA and *t*-test analysis has been conducted to study the influence of these factors on the finger motion and force during eating. The paper is organized as follows: Section 2 presents the method of experimentation employed which is subdivided into two subsections: Experimental Setup and Data Acquisition.  Section 3 presents the data analysis and results obtained while eating different types of food and using different cutlery.  Section 4 presents the discussion of the data analysis results in the previous section, and lastly, the conclusion is drawn in Section 5.

## 2. Experimental Method

### 2.1. Experimental Setup

**Figure 1:**
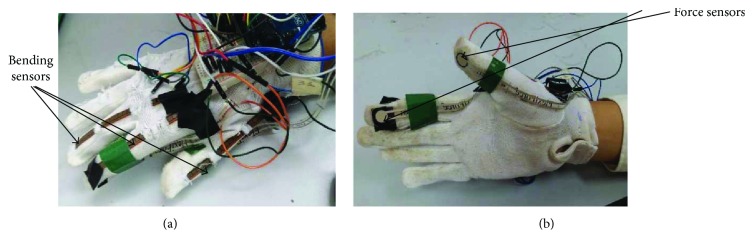
(a) The location of bend sensors on the thumb, middle finger, and index finger. (b) The location of force sensors on the thumb and the index finger.

**Figure 2:**
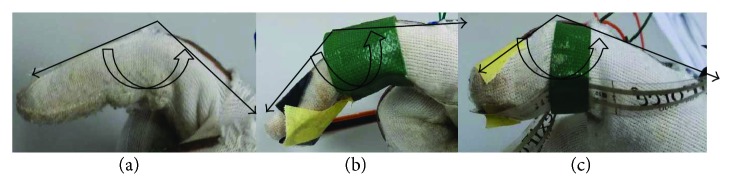
(a) The index finger angle, (b) the middle finger angle, and (c) the thumb angle, measured by the bend sensors.

A prototype glove has been used to analyse the motion of hand during eating (Figure 1). The glove for hand has been designed as an instrument to measure the angle of the index finger, middle finger, and thumb. The glove is developed with three flexible bend sensors (Spectra Symbol, 4.5 inches) for measuring the angles of the index finger, middle finger, and thumb (Figure 2). These bend sensors act as variable resistors which, when flexed, increase the resistance across the sensor. Force sensors (FlexiForce™, A201) are attached to the finger tip of the index and thumb to measure the force exerted by the thumb and index finger, during eating process, since only the index finger and thumb are involved in holding the spoon/fork during any eating activity.

**Figure 3:**
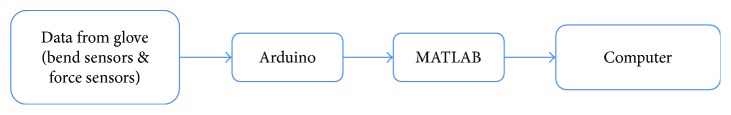
Hardware setup of the bend and force sensors for hand motion analysis.

The data from the glove is recorded using MATLAB 2015 through serial communication with Arduino. (Figure 3) demonstrates the hardware setup of the bend sensors and the force sensors.

### 2.2. Data Acquisition

Six healthy, right-handed subjects including three males and three females, age ranging from 24 to 30 years and an average weight of 65 kgs, volunteered for this study. Five eating activities were performed, to analyse the hand motion, while using different eating cutlery (spoon and fork) and food types (including solids and liquids). The type of food involved in the eating activities included cooked rice, milk cereal, salad with chunks of vegetables, noodles, and a clear soup broth. A plastic spoon and a steel fork were used during the activity. Each activity was performed three times by each participant, with each trial lasting seven seconds and while sitting on a chair with food on the table. The five activities performed were as follows:

1. Eating rice (solid) with a spoon
2. Eating soup broth (liquid) with a spoon
3. Eating cereal with milk (mixture of solid and liquid) using a spoon
4. Eating vegetable salad (solid) using a fork
5. Eating noodles (solid) using a fork

**Figure 4:**
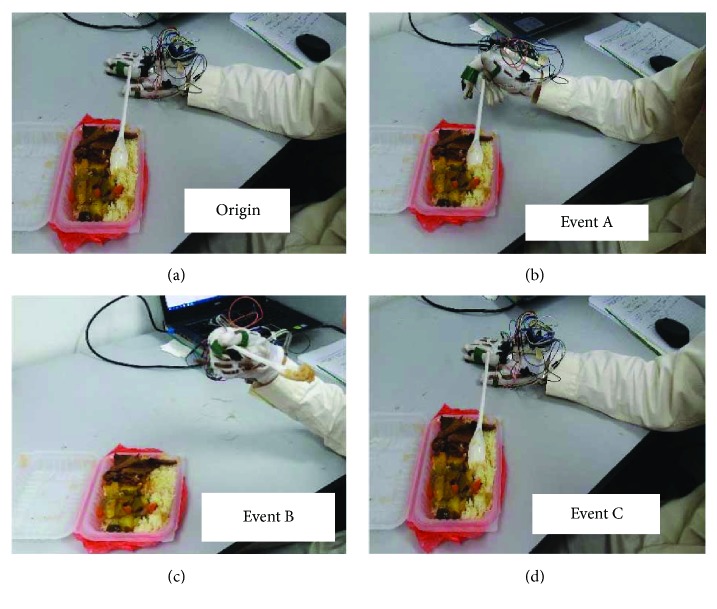
Four main events identified during each eating activity: (a) origin, (b) event A, (c) event B, (d) Event C.

Subjects were trained before performing the activities on how to grasp the cutlery and eat using it, while wearing the glove. For eating noodles, subjects were asked to roll over the noodles on the fork and then eat. Each trial of the eating activities performed consisted of four main events (Figure 4). The first event in each eating activity was the *origin* or starting point, which occurred when the subject kept the glove rested horizontally on the table and bend sensors with almost no bend. The second event called *event A* occurred, when the subject holding a spoon or fork digs in to the food and brings it towards the mouth to eat. The third event known as *event B* occurred, when the subject during eating maintains the grip on the cutlery. The final event is known as *event C*, when the subject releases the cutlery after eating and goes back again to the *origin*, that is, the subject after finishing the eating brought his/her hand back to rest on the table. Throughout the experiment, subjects were asked to keep their elbows rested on the table.

## 3. Data Analysis and Results

### 3.1. Bending Finger Motion Trajectories

**Figure 5:**
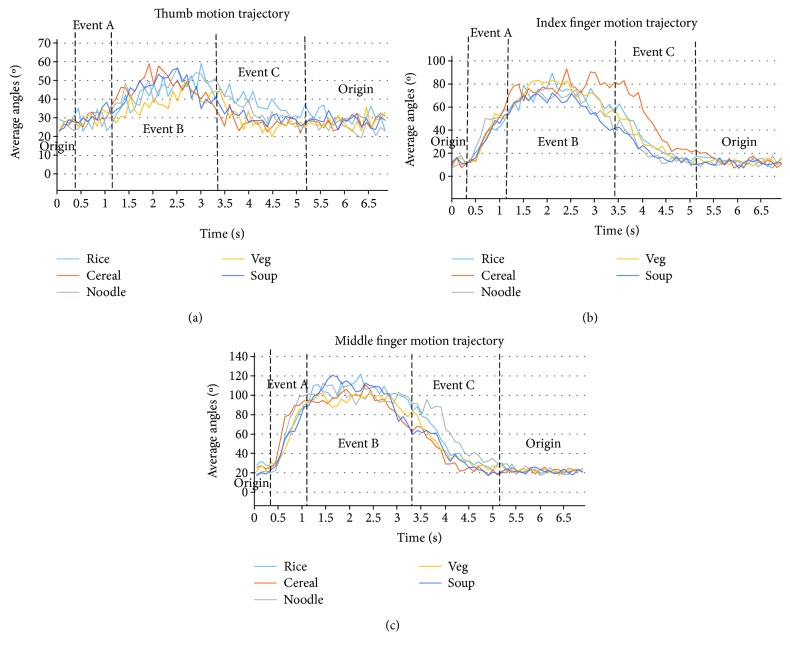
(a) Thumb motion trajectories, (b) index finger motion trajectories, and (c) middle finger motion trajectories obtained from the bend sensor for five different eating activities.

The motion trajectories captured by the bend sensors for the thumb, index finger, and middle finger during five different eating activities are shown (Figure 5) with the four important events identified on the trajectory. During all five eating activities (rice, cereal, and soup with a spoon, noodle and vegetable using a fork), the averaged range of motion (ROM) for the thumb ranged from a minimum of 19.5° to a maximum of 59.1° (referring to Figure 2). The origin is around 19°, although the subjects kept their hands horizontally at rest on the table; this can be due to the sensor fatigue while doing the activities repeatedly (Figure 5(a)).

The ROM of the index finger during eating rice with a spoon is from a minimum averaged angle of 7° to a maximum averaged angle of 90°; for the noodle eating activity, the ROM is from a minimum averaged angle of 7° to a maximum averaged angle of 93°; for cereal with milk activity using a spoon, the ROM is from a minimum averaged angle of 7° to a maximum averaged angle of 75°; for vegetable eating activity using a fork, the ROM is from a minimum averaged angle of 9° to a maximum averaged angle of 83°; and for the soup broth eating activity using a spoon, the ROM is from a minimum of 7° to maximum averaged angle of 72°. The origin in all activities is around 7° (Figure 5(b)).

The ROM of the middle finger whist eating rice with a spoon is from a minimum averaged angle of 18° to a maximum averaged angle of 121.8°; for noodle eating activity, the ROM is from a minimum averaged angle 17° to a maximum averaged angle of 115°; for cereal with milk activity using a spoon, the ROM is from a minimum averaged angle of 17° to a maximum averaged angle of 111°; for eating vegetables using a fork, the ROM is from a minimum averaged angle of 17° to a maximum of 103°; and for the soup broth eating activity using a spoon, the ROM is from a minimum of 17° to a maximum averaged angle of 120.6° (Figure 5(c)).

From the graphs (Figures 5(a)–5(c)), it can be observed that as *event A* starts (at around 0.5 seconds), the bending angles of the fingers start increasing to grip the cutlery and reach a maximum value, while bringing the food to the mouth. During *event B* (lasting around 3 seconds), the magnitude of the angles remains steady, while the subject maintains the grip on the cutlery during eating. The magnitude of the bending angles starts decreasing during *event C* (lasting around 2 seconds), when the subject releases the grip off the cutlery by putting it back into the dish and proceeding towards the *origin*, when the bending angles again settle at around 20°.

### 3.2. Contact Force Trajectories

**Figure 6:**
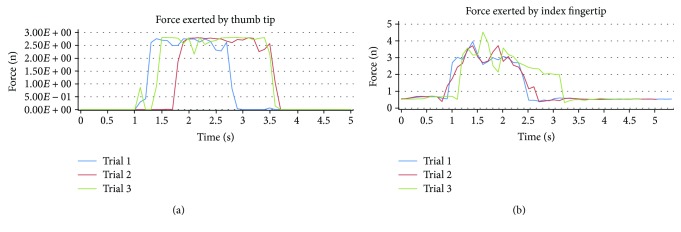
(a) Three trials of thumb-tip force and (b) index fingertip force captured by the force sensor during vegetable eating activity.

The force sensors attached to the prototype glove measured the force exerted by the thumb tip and the index fingertip, while performing five different eating activities, since only the thumb and index finger are involved in holding the spoon or fork while eating. To check the repeatability of the force sensors used, a subject performing three trials of the vegetable eating activity is shown in Figure 6.

**Figure 7:**
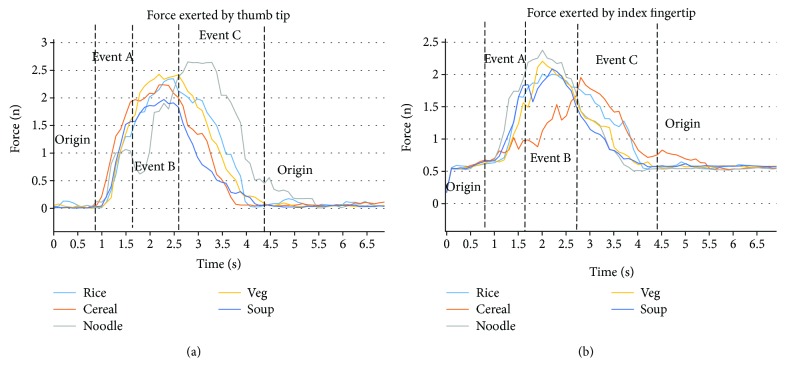
(a) Thumb-tip force and (b) index fingertip force recorded by the force sensor during five different eating activities.

From Figure 6, it can be observed that the force sensor measurements, attached to the index fingertip and the thumb tip, demonstrate quite consistent results.

(Figures 7(a) and 7(b)) demonstrate the force exerted by the thumb tip and the index fingertip with the four main events identified on the graphs. During the *origin*, the hand is lying horizontally on the table, and as such, no force is exerted by the thumb tip/index fingertip, but as *event A* starts and the subject grips a spoon or fork between the thumb and index finger, the magnitude of force increases and during *event C* and a maximum force is reached, when the subject is digging into the food or trying to get the food on the spoon or fork. The force then starts to decrease in *event C*, as the subject is putting the spoon/fork back into the dish, eventually, coming back to the *origin*, when the subject rests his/her hand on table again. During the noodle eating activity using a fork, the thumb tip exerts a maximum average force of 2.7 N, which is the highest of all other eating activities. Since during the noodle eating activity, the subjects were asked to roll over the noodles on the fork; hence, the force trajectory shows some minor fluctuations and is longer than other eating activities. For rice eating activity using a spoon, a maximum average force of 2.4 N; for cereal with milk eating activity using a spoon, a maximum average force of 2.2 N; for vegetable eating activity using a fork, a maximum average force of 2.4 N; and for soup broth eating activity using a spoon, a maximum average force of 2.0 N is exerted by the thumb tip (Figure 7(a)).

During the cereal with milk eating activity using a spoon, the index fingertip exerts a maximum average force of 2.4 N, which is the highest of all other eating activities. For rice eating activity using a spoon, a maximum average force of 2.0 N; for noodle eating activity using a fork, maximum average force of 2.0 N; for vegetable eating activity using a fork, a maximum average force of 2.2 N; and for soup broth eating activity using a spoon, a maximum average of 2.1 N force is exerted by the index fingertip (Figure 7(b)).

### 3.3. Correlations between Bending Angles and the Contact Forces of Fingers

The bending finger angle data and the contact force data captured by the sensors has been used to find the correlations between the bending angles of the thumb, index finger, and middle finger and the contact forces exerted by the thumb and index finger, during eating activities using the *Pearson product moment correlation coefficient* (PPMC). Pearson correlation coefficient (*r*) measures the strength and direction of a linear relationship between two variables. The SPSS statistics software package has been used to perform this analysis. It takes values ranging from +1 to −1; *r* *= +*1 implies a strong positive linear relationship between the variables, while *r* = 1 implies a strong negative linear relationship, and *r* = 0 implies no linear relationship between the variables. Equation (1) gives the formula for computing the Pearson correlation coefficient [20].

(1) $$\begin{array}{c} r=\frac{n\sum \left(xy\right)-\left(\sum x\right)\left(\sum y\right)}{\sqrt{\left\{n\left(\sum x^{2}\right)-\left(\sum x^{2}\right)\right\}\left\{n\left(\sum y^{2}\right)-\left(\sum y^{2}\right)\right\}}}, \end{array}$$

where *n* = number of data pairs.

The averaged Pearson coefficients of different bending finger angles and the forces exerted by the index finger and thumb, for all six subjects involved in the experiment, are shown in (Table 2).

The most significant coefficients are highlighted (Table 2). From the results (Table 2), both the soup (liquid) and cereal (solid and liquid) eating activities which are performed using a spoon have similar results, where both index fingertip force and the thumb-tip force have shown the strongest positive linear relationship with the average bending motion of the thumb. Noodle eating activity has shown similar results with the soup and cereal activities. Moreover, during the vegetable and rice eating activities, the force exerted by the thumb tip and the index fingertip have the strongest positive correlation with the averaged index finger motion. During all the eating activities, the middle finger motion has the weakest linear relationship with the index fingertip and thumb-tip force as compared to the bending motion of the index finger and thumb.

### 3.4. One-Way ANOVA of the Bending Angles of Fingers with Respect to Different Types of Food

The one-way analysis of variance or ANOVA is a statistical comparison test used to determine whether there are any statistically significant differences between the means of two or more independent groups. SPSS Statistics software package has been used to perform ANOVA in this study. In this study, ANOVA has been used to determine whether the bending motion of the index finger (*BENDINDX*), middle finger (*BENDMID*), and thumb (*BENDTHMB*) differed based on different groups of food types (cereal, rice, vegetable, noodle, and soup) or not (Tables 3 and 4).

The df column in Table 3 means degrees of freedom, which is the division of *Sum of squares* by *Mean square* values in the ANOVA summary table. The *Sum of squares* is the sum of *Between groups* and *Within groups*. The *Sig.* column denotes the *p* value which represents the probability of finding an effect equal to or greater than the one observed considering the null hypothesis to be true. The null hypothesis here signifies that there is no significant difference in the bending angles of the thumb, index finger, and middle finger with respect to different types of food groups.

The lower the *p* value, the more likely the null hypothesis is rejected (preferably less than 0.05, while 0.10 is also accepted but as a weak evidence). The *p* value thus provides a quantitative strength of evidence against the null hypothesis stated [21, 22]. From (Table 3), it can be concluded that for the average bending motion of the thumb and the index finger across the five groups of food (soup, rice, noodle, cereal, and vegetable salad), there is a statistically significant difference at 5% and 10%, respectively, whereas for the averaged bending motion of the middle finger, there is no statistically significant difference, while eating different types of food (*p* > 10%). In simple words, it can be concluded from the ANOVA results that the bending motion of the thumb and the index finger is influenced by the type of food, whereas the bending motion of the middle finger is not affected by type of food.

An LSD (least significant difference) post hoc test has been carried out to distinguish eating precisely which type of food group (noodle, soup, rice, vegetables, and cereal) the variances occur in the bending motions of the thumb and the index finger (Table 4). To check for the variances, *Mean difference (I-J)* and *Sig.* (significance/*p* value) column of Table 4 is considered. The LSD results for the bending angles of the thumb from Table 4 can be summarised as follows:

1. The bending angles of thumb during cereal eating activity are smaller than its bending angles during rice eating activity at a statistically significant difference of 10% (*p* = 0.075, read row 1).
2. On the contrary, the bending angles of thumb during vegetable, noodle, and soup eating activities show statistically no significant difference with the bending motion of thumb during cereal eating activity. In other words, the bending motion of the thumb during vegetable, noodle, and soup eating activities does not show any variance with respect to the cereal eating activity.
3. The bending angles of thumb during rice eating activity are again greater than its bending angles during the vegetable eating activity, at a statistically significant difference of 1%, while the bending angles of thumb during noodle and soup activities show statistically no significant difference with the bending motion of thumb during rice eating activity.
4. Similarly, the bending angles of thumb during vegetable eating activity are lesser than its bending angles during noodle and soup eating activities at a statistically significant difference of 10% and 5%, respectively.
5. Finally, the bending motion of the thumb during noodle eating activity has statistically no significant difference with the bending motion of the thumb during soup eating activity.

Therefore, from the LSD post hoc results, it can be concluded that the bending motion of the thumb during rice and vegetable eating activity is maximum as compared to other eating activities and the bending motion of the thumb does not show much variance during noodle and soup eating activities.

Similarly, the ANOVA results for bending angles of index finger (Table 4) can be summarised as follows:

1. The bending angles of index finger during cereal eating activity are smaller than its bending angles during the noodle eating activity, at a statistically significant difference of 5%.
2. Contrary, the bending angles of index finger during rice, vegetable, and soup eating activity show statistically no significant difference with the bending motion of index finger during cereal eating activity.
3. The bending angles of the index finger during rice eating activity show statistically no significant difference with the bending motion of the index finger during the vegetable, noodle, and soup eating activities.
4. During the vegetable eating activity, the bending motion of the index finger shows statistically no significant difference with the bending motion of the index finger during noodle and soup eating activities.
5. Finally, during the noodle eating activity, the bending angles of the index finger are greater than its bending angles, during soup eating activity at a statistically significant difference of 10%.

Thus, from the LSD post hoc test results, it can be summarised that the bending motion of the index finger during noodle eating activity is highest as compared to the other four eating activities. Additionally, the bending motion of the index finger does not show any significant variance with respect to soup, vegetable, and rice eating activities.

### 3.5. One-Way ANOVA of the Forces Exerted by the Finger Tips with Respect to Different Types of Food

The one-way ANOVA technique has been again used to determine if there exists a statistically significant difference in the averaged forces exerted by the thumb tip (*FTHMB*) and the index fingertip (*FINDX*), based on the different groups of food type (Table 5). From the ANOVA results, it can be concluded that for both forces exerted by the thumb and the index finger, there exists statistically no significant difference during various eating activities (*p* > 10%). As seen from the *Sig*. column in Table 5, for both index finger and the thumb, the *Sig.* is 0.892 (89.2%) and 0.273 (27.3%), respectively, which is far greater than the desired *Sig*. or *p* value of less than 10%. That is, the contact forces of the thumb tip and the index fingertip are not influenced by the type of food to be consumed.

### 3.6. An Independent Samples *t*-Test of Bending Angles of Fingers with Respect to Different Eating Tools

An independent samples *t*-test has been conducted using SPSS software to find whether the averaged bending angles of the thumb, index finger, and middle finger vary with respect to two different eating tool groups (a fork and a spoon). In this case, *t*-test is conducted instead of ANOVA because here, the *factor* (independent variable) which is the cutlery has only two groups (fork/spoon), but for conducting ANOVA, it must be more than two; hence, an independent samples *t*-test has been conducted.

The results of the independent samples *t*-test are shown in Tables 6 and 7. To interpret the results from the *t*-test table (Table 7), the large column labelled *Levene's test for equality of variances* is checked first. This is a test that determines if the two conditions (a fork and a spoon) have about the same or different amounts of variability between scores. Under this column, the *Sig. p* value column is considered. This *Sig*. value determines which row to consider, either the *Equal variances assumed* or the *Equal variances not assumed* row. If the *Sig*. value is *greate*r than 0.05, read from the top row, which means that the variability in the two conditions is about the same. That is, the scores in one condition (fork) do not vary much more than the scores in the second condition (spoon). Put scientifically, it means that the variability in the two conditions is not significantly different and vice versa, if the *Sig.* value is lesser or equal to 0.05. In the latter case, read from the bottom row, that the variability in the two conditions is not the same. That is, the scores in one condition vary much more than the scores in the second condition. Scientifically, it means that the variability in the two conditions is significantly different.

From Table 7, for the bending motion of the index finger (*BENDINDX*), the *p* value is less than 0.05 (0.000), that is, reading from the bottom row, which reveals that the variability in the two conditions (fork and spoon) is not the same. After finding the row to read (bottom row), now the results of *t*-test can be found in the column labelled *t-test for equality of mean* by considering the *Sig. (2-tailed)* column under it. This *Sig. (2-tailed)* column determines if the two conditions' means are statistically different. If the *Sig. (2-tailed)* is greater than 0.05, this means that there is no statistically significant difference between the two conditions. That is, the variances between condition means are likely due to chance and not likely due to the factor (independent variable) manipulation and vice versa for *Sig. (2-tailed)* lesser or equal to 0.05. From Table 7, for the bending motion of the index finger (*BENDINDX*), the *Sig. (2-tailed)* value is *0.034*, which is less than 0.05. Thus, it can be concluded that there exists a statistically significant difference in the means of bending motion of the index finger, while using a fork (mean = 41.392) and a spoon (mean = 35.19) condition. That is, the bending motion of the index finger is influenced by the type of cutlery used. Since results from (Table 6 showed that the mean of a fork condition is higher than that of the spoon condition, it can be concluded that the bending angles of the index finger using a fork are greater than its bending angles, while eating with a spoon.

Similarly, for the bending motion of the middle finger (*BENDMID*), the results reveal that there exists no statistically significant difference, whether eating with a spoon or a fork; hence, the bending angles of the middle finger remain unaffected, irrespective of the cutlery used (*Sig. (2-tailed = 0.609)*, reading from the top row).

The *t*-test results for the bending motion of the thumb (*BENDTHMB*) (Tables 6 and 7) showed that there also exists a statistically significant difference in the means of bending motion of the thumb, while using a fork (mean = 33.02) and a spoon (mean = 34.97) condition (*Sig. (2-tailed = 0.045)*, reading from the top row). The results from (Table 6) showed that the mean of a spoon condition is higher than that of the fork condition; it can be concluded that the bending angles of thumb using a spoon are greater than its bending angles while eating with a fork.

### 3.7. An Independent Samples *t*-Test of the Contact Forces Exerted by the Fingertips while Using Different Cutlery

A similar independent samples *t*-test (Tables 8 and 9) has been performed to find if the average contact forces exerted by the thumb tip and the index fingertip vary with respect to different eating tool groups (a fork and a spoon). Following the same rules of interpreting the *t*-test as in the previous section, it can be concluded from Tables 8 and 9 that for the average contact force exerted by the index finger (*FINDX*), there exists statistically no significant difference in the means of the two conditions; that is, the contact force exerted by the index finger is not influenced whether a fork or a spoon is used (*Sig. (2-tailed) = 0.494*, reading from the bottom row). Similar results have been obtained for the average contact force exerted by the thumb (*FTHUMB*); that is, the contact force exerted by the thumb is not influenced whether a fork or a spoon is used (*Sig. (2-tailed) = 0.118*, reading from the bottom row).

## 4. Discussion

Pearson correlation coefficient has been used to establish a relationship between the bending motion of the thumb, index finger, and middle finger and the contact forces exerted by the thumb tip and the index fingertip during different eating activities. The results revealed that for the cereal and soup eating activity using a spoon, the correlation coefficients showed the same trend with the thumb motion, having the strongest positive correlation with the index fingertip force and thumb-tip force, respectively. This can be attributed to the fact that since, both the activities involve similar eating action using a spoon, with only the food characteristics being different. Noodle and vegetable eating activities using a fork showed different results. This can be due to the different eating action involved while picking up the food as the noodle involved rolling action of the fork. From the correlation results, it can also be concluded that the middle finger motion has the weakest positive linear relationship with the index fingertip and the thumb-tip force during all five eating activities, irrespective of the eating tools and food characteristics as compared to the thumb and index finger bending motion.

A one-way ANOVA test has been done to compare the bending motion of the thumb, index finger, and the middle finger and the contact forces exerted by the thumb and the index finger while eating different food types (cooked rice, cereal with milk, vegetable salad, soup broth, and noodles). It can be concluded that the bending angles of thumb during the rice eating activity are relatively greater than cereal and vegetable eating activities. Additionally, the bending motion of the thumb during the vegetable eating activity is relatively smaller than its bending motion during the noodle and soup eating activity. Regarding the bending motion of the index finger, in all five eating activities, it can be concluded that during the noodle eating activity, the bending angles are relatively greater than cereal and soup eating activities, while the bending angles of the index finger show no significant statistical difference during the rice and vegetable eating activities. The ANOVA results also revealed that the bending motion of the middle finger showed no significant statistical difference to different types of food. This means that the bending motion of the middle finger is not varied much by food characteristics (solid or liquid or mixture of solid and liquid). The ANOVA results for the contact forces exerted by the thumb and the index finger show that the forces are unaffected by the type of food.

An independent samples *t*-test has been carried out to compare the bending angles of the thumb, index finger, and middle finger and the contact forces exerted by the thumb and the index finger, respectively, while using different cutlery (fork and spoon). The results revealed that the bending angles of the index finger and the thumb are influenced by the type of cutlery unlike the middle finger which remains unaffected. Thus, we can conclude that the motion of the middle finger is not affected by the type of food characteristics and the types of cutlery (fork/spoon) being used. The independent samples *t*-test also revealed that the contact force exerted by the thumb and the index finger is not influenced by the cutlery. Hence, it can be concluded that the contact force exerted by the index fingertip and the thumb tip are not influenced by the different food characteristics nor by the cutlery being used.

## 5. Conclusion

In this study, human hand motion analysis was carried out on five different eating activities with six subjects. The ANOVA and *t*-test results revealed that the bending motion of the index finger and the thumb is affected with respect to different food characteristics as well as the type of cutlery used, that is, a fork and a spoon, whereas the bending motion of the middle finger remains unaffected. In addition, the contact force exerted by the thumb tip and index fingertip remains unaffected with respect to the different types of food and cutlery used. These results can be useful in the future to differentiate hand motions dependent on different eating activities and the different cutlery (fork/spoon) used. It can further be used in the development of a mathematical model of the hand for eating rehabilitation purposes.

## Figures and Tables

**Table 1:** Highlights of the previous contributions to human motion analysis.

Number	Authors	Objective	Focus of study	Data acquisition method	Results/findings	Activity
1	Ju and Liu [11]	To correlate the muscle signals with contact forces and finger trajectories & motion recognition using muscle signals	Human hand motion analysis with multisensory information	EMG sensor, force sensor & DataGlove	Strong correlations between muscle signals, contact forces, and finger trajectories. Fuzzy Gaussian mixture models (FGMMs) used for motion recognition	Ten in-hand manipulations like holding & lifting a dumbbell

2	Gopura et al. [12]	To analyse upper-limb muscle activities during basic upper-limb motion, to design power-assist robotic exoskeleton systems	Human upper-limb muscle activities during daily upper-limb motions	EMG electrodes, VICON motion capture system	Relationships between the upper limb motions & activity levels of main muscles have been established	Basic motions and the selected daily activities of upper-limb

3	Tang et al. [13]	To classify multiple hand gestures using three different methods	Hand motion classification using a multichannel surface sEMG sensor	sEMG sensors	Experimental results showed that the success rate for the identification of all the 11 gestures is significantly high	11 hand gestures

4	Cabibihan et al. [14]	To analyse the gesture, the amount of force applied on regions of the hand, and the angular motion of finger joints	Human patting gesture analysis for robotic social touching	CyberGlove II FingerTPS sensors	The sensitive regions on the hand while performing pat have been identified	Human patting gesture

5	Rosen et al. [15]	To study the kinematics and the dynamics of the human arm during daily activities	The human arm kinematics and dynamics during daily activities	VICON motion capture system & reflective markers	The results indicated that the various joints' kinematics and dynamics change significantly based on the nature of the task	24 ADL

6	Ah et al. [16]	To evaluate motor control abilities between the groups of people with mild and moderate arm impairments	3D kinematic motion analysis of door handling task in people with mild and moderate stroke	VICON motion capture system & reflective markers	Comparisons have been drawn between healthy, mild & moderate stroke patients	Door handling task

7	Aprile et al. [17]	To analyse, using motion analysis, the qualitative and quantitative upper limb motor strategies in stroke patients	Kinematic analysis of the upper limb motor strategies in stroke	Smart motion capture optoelectronic system	Comparisons have been drawn between stroke & healthy control group while reaching out for the glass to drink	Drinking task

8	Adnan et al. [18]	To develop a low-cost DataGlove, able to recognize the different finger activities	Measurement of the flexible bending force of the index and middle fingers for virtual interaction	Low-cost DataGlove by using the flexible bending sensor	The DataGlove developed can measure several human degrees of freedom (DoFs)	Sign language translation (letters A, B, C, D, F & K and number 8)

9	Adnan et al. [19]	To find the correlations between the forces of finger phalanges	Accurate measurement of force by the force sensor for intermediate and proximal phalanges of index finger	Flexiforce pressure sensors	An analytical mathematical model and ANOVA has been established to predict the force induced at the flexible force sensor and the human finger of low-cost DataGlove	Any finger gripping activity

**Table 2:** Averaged Pearson coefficient of bending finger angles and force exerted by fingers.

	Rice (spoon)			Cereal & Milk (spoon)			Soup (spoon)			Vegetable (fork)			Noodle (fork)		
	Index	Middle	Thumb	Index	Middle	Thumb	Index	Middle	Thumb	Index	Middle	Thumb	Index	Middle	Thumb
FINDEX	**0.93**	0.87	0.78	0.89	0.84	**0.95**	0.90	0.87	**0.94**	**0.89**	0.81	0.74	0.85	0.73	**0.89**
FTHUMB	**0.90**	0.84	0.81	0.89	0.83	**0.94**	0.90	0.89	**0.94**	**0.90**	0.83	0.80	0.86	0.72	**0.88**

**Table 3:** Analysis of variance summary table.

		Sum of squares	df	Mean square	Sig./*p* value
BENDTHMB	Between groups	893.33	4	223.33	0.023
	Within groups	26855.55	345	77.84	
	Total	27748.88	349		

BENDINDX	Between groups	5765.47	4	1441.37	0.074
	Within groups	230984.22	345	669.52	
	Total	236749.69	349		

BENDMID	Between groups	3236.77	4	809.19	0.609
	Within groups	413100.47	345	1197.39	
	Total	416337.24	349		

**Table 4:** A least significant difference post hoc test using SPSS software package.

Dependent variable	(*I*) foodtype	(*J*) foodtype	Mean difference (*I* − *J*)	Sig.
BENDTHMB				
1	Cereal	Rice	−2.66	0.075
		Veg	2.33	0.119
		Noodle	−0.60	0.687
		Soup	−0.61	0.684
2	Rice	Cereal	2.66	0.075
		Veg	4.99^∗^	0.001
		Noodle	2.06	0.168
		Soup	2.06	0.169
3	Veg	Cereal	−2.33	0.119
		Rice	−4.99^∗^	0.001
		Noodle	−2.93	0.050
		Soup	−2.94^∗^	0.050
4	Noodle	Cereal	0.60	0.687
		Rice	−2.06	0.168
		Veg	2.93	0.050
		Soup	0.00	0.998
5	Soup	Cereal	0.61	0.684
		Rice	−2.06	0.169
		Veg	2.94^∗^	0.050
		Noodle	0.00	0.998

BENDINDX				
6	Cereal	Rice	−3.29	0.452
		Veg	−3.33	0.447
		Noodle	−9.97^∗^	0.023
		Soup	1.96	0.655
7	Rice	Cereal	3.29	0.452
		Veg	−0.04	0.993
		Noodle	−6.68	0.127
		Soup	5.25	0.231
8	Veg	Cereal	3.33	0.447
		Rice	0.04	0.993
		Noodle	−6.65	0.129
		Soup	5.28	0.228
9	Noodle	Cereal	9.97^∗^	0.023
		Rice	6.68	0.127
		Veg	6.65	0.129
		Soup	11.93^∗^	0.007
10	Soup	Cereal	−1.96	0.655
		Rice	−5.25	0.231
		Veg	−5.28	0.228
		Noodle	−11.93^∗^	0.007

^∗^The mean difference is significant at the 0.05 level.

**Table 5:** ANOVA summary table.

		Sum of squares	df	Mean square	Sig./*p* value
FINDX	Between groups	0.30	4	0.08	0.892
	Within groups	94.05	345	0.27	
	Total	94.35	349		

FTHMB	Between groups	3.54	4	0.88	0.273
	Within groups	236.09	345	0.68	
	Total	239.63	349		

**Table 6:** Group statistics showing the mean and standard deviation (SD) for the bending motion data analysis.

	Cutlery	Mean	Std. deviation
BENDINDX	Fork	41.39	28.29
	Spoon	35.19	24.19

BENDMID	Fork	58.12	33.62
	Spoon	56.19	35.20

BENDTHMB	Fork	33.02	8.81
	Spoon	34.97	8.92

**Table 7:** Independent samples *t*-test results for the bending motion data analysis.

		Levene's test for equality of variances		*t*-test for equality of means		*t*-test for equality of means
		*F*	Sig.	*t*	df	Sig. (2-tailed)
BENDINDX	Equal variances assumed	13.38	0.000	2.20	348	0.029
	Equal variances not assumed			2.13	265.58	0.034

BENDMID	Equal variances assumed	0.22	0.643	0.51	348	0.609
	Equal variances not assumed			0.52	307.26	0.606

BENDTHMB	Equal variances assumed	0.11	0.742	−2.02	348	0.045
	Equal variances not assumed			−2.02	300.69	0.044

**Table 8:** Group statistics showing the Mean and SD for the contact force data analysis.

	Cutlery	Mean	Std. deviation	Std. error mean
FINDX	Fork	0.90	0.46	0.04
	Spoon	0.94	0.55	0.04

FTHMB	Fork	0.74	0.90	0.08
	Spoon	0.60	0.77	0.05

**Table 9:** Independent samples *t*-test results for the contact force data analysis.

		Levene's test for equality of variances		*t*-test for equality of means		*t*-test for equality of means
		F	Sig.	*t*	df	Sig. (2-tailed)
FINDX	Equal variances assumed	9.60	0.002	−0.66	348.00	0.509
	Equal variances not assumed			−0.68	330.37	0.494

FTHMB	Equal variances assumed	5.74	0.017	1.62	348.00	0.106
	Equal variances not assumed			1.57	265.59	0.118
